# REM Sleep Behaviour Disorder in Multiple System Atrophy: From Prodromal to Progression of Disease

**DOI:** 10.3389/fneur.2021.677213

**Published:** 2021-06-14

**Authors:** Giulia Giannini, Federica Provini, Pietro Cortelli, Giovanna Calandra-Buonaura

**Affiliations:** ^1^IRCCS Istituto delle Scienze Neurologiche di Bologna, Unità Operativa Complessa (UOC) Clinica Neurologica Rete Metropolitana NEUROMET, Bologna, Italy; ^2^Department of Biomedical and NeuroMotor Sciences (DiBiNeM), Alma Mater Studiorum - University of Bologna, Bologna, Italy

**Keywords:** disease progression, sleep disorders, phenoconversion, prodromic phase, review, autonomic failure, REM sleep behaviour disorder, multiple system atrophy

## Abstract

A higher frequency of motor and breathing sleep-related disorders in multiple system atrophy (MSA) populations is reported. REM sleep behaviour disorder (RBD) is one of the most robust markers of an underlying alpha-synucleinopathy. Although a large corpus of literature documented the higher prevalence of RBD in MSA, few studies have systematically investigated the prevalence of RBD as mode of disease onset and its role in disease progression. Moreover, there has been increasing interest in phenoconversion into synucleinopathies of cohorts of patients with isolated RBD (iRBD). Finally, some studies investigated RBD as predictive factor of conversion in isolated autonomic failure, a synucleinopathy presenting with autonomic failure as the sole clinical manifestation that could convert to a manifest central nervous system synucleinopathy. As the field of neurodegenerative disorders moves increasingly towards developing disease-modifying therapies, detecting individuals in the prodromal stage of these synucleinopathies becomes crucial. The aims of this review are to summarise (1) the prevalence of RBD during the course of MSA and as presenting feature of MSA (iRBD), (2) the RBD features in MSA, (3) MSA progression and prognosis in the subgroup of patients with RBD predating disease onset, and (4) the prevalence of MSA conversion in iRBD cohorts. Moreover, we summarise previous results on the role of RBD in the context of isolated autonomic failure as marker of phenoconversion to other synucleinopathies and, in particular, to MSA.

## Introduction

Multiple system atrophy (MSA) is a progressive neurodegenerative disorder characterised by a heterogeneous combination of autonomic failure, cerebellar syndrome, parkinsonian features poorly responsive to levodopa, and pyramidal signs (1).

The diagnostic criteria define three degrees of certainty for diagnosis (possible, probable, and definite) and two phenotypes: parkinsonian (MSA-P) or cerebellar (MSA-C), according to the predominant feature at the time of evaluation (1). Disease onset is currently defined as the initial presentation of any motor sign whether parkinsonian or cerebellar, or autonomic features with the exception of male erectile dysfunction (1).

Sleep disorders are frequently seen in MSA populations. Some of these overlap across the other parkinsonisms, while others are either unique to or far more prevalent in this disease. Sleep disorders in MSA are multifactorial and include nocturnal and diurnal manifestations. Nocturnal sleep is characterised by reduced sleep efficiency, fragmented sleep, and frequent awakenings, resulting in poor sleep quality. These disturbances are due to the neurodegeneration itself, linked to motor (akinesia and rigidity) and autonomic symptoms (nocturia) related to the disease, but also to the presence of concomitant sleep motor or behaviour disorders such as REM sleep behaviour disorder (RBD), restless legs syndrome (RLS), periodic limb movements (PLMs), or sleep-related breathing disorders (2–5). Few studies reported diurnal manifestations including excessive daytime sleepiness, evaluated through questionnaires, and spontaneous or levodopa-induced sleep attacks, but these results were not confirmed in other studies (5–9).

RBD is a parasomnia characterised by loss of the physiological muscle atonia during REM sleep associated with complex, sometimes violent, and dangerous motor behaviours during which patients act out the content of their dreams (10). Although RBD is readily clinically suspected, video-polysomnography (V-PSG) is required to demonstrate REM sleep without atonia (RSWA) and, therefore, to confirm the diagnosis. The presence of RBD could be over- or underestimated when based only on clinical history. On one side, different sleep disorders may mimic RBD (obstructive sleep apnoea, somnambulism, nocturnal epilepsy, hallucinations, confusional arousals, and RLS with PLMs), and on the other side, a proportion of patients are unaware of their abnormal behaviours during the night (11).

RBD is one of the most robust markers of an underlying alpha-synucleinopathy, but its presence does not distinguish MSA from other alpha-synucleinopathies like Parkinson's disease (PD) and dementia with Lewy bodies (DLB) (12, 13). Moreover, RBD could predate the motor/autonomic onset of these diseases (12, 13). Although a large corpus of literature documented the higher prevalence of RBD in MSA, few studies have systematically investigated the prevalence of RBD as mode of disease onset and its role in disease progression.

In recent years, there has been increasing interest in cohorts of patients with isolated RBD (iRBD) aiming to calculate the rate and latency of phenoconversion into synucleinopathies and to detect clinical and instrumental features acting as reliable markers of these neurodegenerative subtypes (MSA, PD, DLB) (12, 13). Some studies investigated RBD as predictive factor of conversion into isolated autonomic failure (IAF), a synucleinopathy presenting with autonomic failure as the sole clinical manifestation that could convert to a manifest central nervous system synucleinopathy (14–16). As the field of neurodegenerative disorders moves increasingly towards developing disease-modifying therapies, detecting individuals in the prodromal stage of these synucleinopathies becomes crucial. Identifying specific features predicting a particular subtype of these neurodegenerative diseases in cohorts of iRBD patients remains, to date, a challenge.

The aims of this narrative review are to summarise the available literature on (1) the prevalence of RBD during the course of MSA and as presenting features of MSA, (2) the RBD features in MSA, (3) MSA progression and prognosis in the subgroup of patients with RBD predating disease onset, and (4) the prevalence of MSA conversion in iRBD cohorts. Finally, we summarise previous results on the role of RBD in the context of IAF as marker of phenoconversion to other synucleinopathies and, in particular, to MSA.

Publications in English language issued before July 2020 were initially searched in PubMed database by using the following search criteria: [(“Shy-Drager”) OR (“multiple system atrophy”) OR (“striatonigral degeneration”) OR (“olivopontocerebellar”) OR (“MSA”) OR (“autonomic failure”) OR (“synucleinopathies”)] AND [(Sleep) OR (REM sleep behaviour disorder) OR (RBD) OR (Isolated RBD) OR (Idiopathic RBD) OR (REM sleep without atonia)]. In addition, a manual search of bibliographies of included studies and related reviews was carried out to find additional references. No unpublished data or data from abstracts were encountered or used. A subsequent review process with update of the literature search was performed between August 2020 and January 2021.

## REM Sleep Behaviour Disorder in MSA

### Prevalence of RBD in MSA

Eighteen prospective and retrospective studies (7, 17–33), most of which on a small sample, investigated the prevalence of clinically suspected RBD in MSA during the disease course (Table 1). Sixteen articles included the prevalence of RBD confirmed by PSG, 13 of them monitored with synchronised audiovisual recording (V-PSG) (Table 1). A meta-analysis performed in 2015 on 13 studies reported a summary prevalence of clinically suspected RBD in MSA ranging from 25 to 100%, with a summary prevalence of 73% (95% confidence interval [CI] = 62–84%) in a pooled sample of 324 patients. The prevalence of PSG-confirmed RBD in MSA ranged from 68.8 to 100%, with a summary prevalence of 88% (95% CI = 79–94%) in the pooled sample (30). Other five studies (27, 28, 31–33), not included in the meta-analysis, reported a prevalence of clinically suspected RBD ranging from 44.4 to 76.6% and of V-PSG-confirmed RBD ranging from 32.7 to 76.0% (Table 1).

**Table 1:** Studies focused on the prevalence of clinically suspected or V-PSG-confirmed RBD in MSA patients.

**References**	**Study sample**	**Patients who underwent V-PSG**	**Clinically suspected RBD**	**V-PSG-confirmed RBD**
Plazzi et al. (17)	39	39	69.2%	89.7%
Tachibana et al. (18)	21	21	–	90.5%
Iranzo et al. (19)	20	20	90.0%	90.0%
Silber and Levine (20)	28	28	60.7%	71.4%
Wetter et al. (21)	10	10	–	70%
Boeve et al. (22)	13^*^	10^*^	84.6%	90.0%
Ghorayeb et al. (7)	57	–	47.5%	–
Vetrugno et al. (23)	19	19	78.9%	100.0%
Schmeichel et al. (24)	11^*^	5^*^	72.7%	100.0%
De Cock et al. (25)	49	19	87.8% (91.7% MSA-C, 84.0% MSA-P)	100.0%
Nomura et al. (26)	16^*^	16^*^	25.0%	68.8%
Muntean et al. (27)	36	36	–	72.7% MSA-C, 76.0% MSA-P
Guo et al. (28)	30	30	46.7% (52.2% MSA-C, 28.6% MSA-P)	46.7%
Stanzani-Maserati et al. (29)	10	10	–	100%
Palma et al. (30)	42	42 (2 had no REM during the study)	76.2%	81.0% (77.8% MSA-C, 86.7% MSA-P)
Coon et al. (31)	685	–	44.4% (48.6% MSA-C, 41.9% MSA-P)	–
Giannini et al. (32)	158	121	76.6%	67.7%
Wang et al. (33)	55	55		32.7% (50.0% MSA-C, 25.6% MSA-P)

*These prevalence rates were calculated from raw data, when not available in the studies*.

* *Studies investigating RBD by using PSG (without synchronized audiovisual recording); MSA-C, multiple system atrophy with predominant cerebellar phenotype; MSA-P, multiple system atrophy with predominant parkinsonism; PSG, polysomnography; RBD, REM sleep behaviour disorder; V-PSG, video-polysomnography*.

To note, several studies reported RBD diagnosis as video-polysomnographic finding also in MSA patients with a negative symptomatic history for this sleep disorder (17, 18, 26, 30, 34).

### RBD as Initial Manifestation of MSA

In patients with synucleinopathies, RBD frequently predates motor and cognitive deficits (11, 12). Despite its higher prevalence in MSA, the prevalence of RBD as initial manifestation of disease has been poorly investigated.

Few studies primarily investigated RBD as symptom of MSA onset. However, these data were reported also in other studies focused on different aims. To date, 12 studies have reported RBD preceding the clinical MSA onset (17, 19, 23, 25, 26, 28–32, 34, 35) (Table 2). Prevalence of RBD as first symptom of disease onset ranged from 10 to 60.0% when calculated in overall MSA samples and from 21.4 to 63.6% when calculated only in MSA patients with RBD (Table 2). This variability in results may be a consequence of the differences in study design, sample size, population characteristics, follow-up duration, and diagnostic criteria used to determine RBD. The first cause of heterogeneity derived from study design and population characteristics because the majority of the studies included all MSA patients, while other focused on RBD characteristics and, for this reason, included only MSA patients with clinically suspected RBD. Another cause of this variability is related to the sample size: the majority of studies included <50 patients (17, 19, 23, 25, 26, 28, 29, 34, 35), one study included between 50 and 100 patients (30), and two studies included >100 patients (31, 32). Moreover, in eight studies, RBD was diagnosed with V-PSG (17, 19, 23, 26, 28, 29, 32, 34), while in the other four studies (25, 30, 31, 35) the prevalence of iRBD was calculated on clinically suspected RBD because RBD diagnosis was based on history taking (31), V-PSG was performed only in a subgroup of the iRBD sample (35), or the rate of V-PSG-confirmed RBD in patients presenting with RBD was not specified in the results section (25, 30).

**Table 2:** Studies reporting isolated RBD as initial manifestation of MSA.

**References**	**Study sample**	**V-PSG**	**Prevalence of RBD preceding autonomic/motor onset in MSA patients with RBD**	**Prevalence of RBD preceding autonomic/motor onset in overall study sample**	**Latency from iRBD onset to first MSA symptom**
Plazzi et al. (17)	39	Yes	44.4% (12/27)	30.8% (12/39)	From 19 to 1 years
Iranzo et al. (19)	20	Yes	33% (6/18)	30% (6/20)	From 20 to 7 years, mean 13.4 years (SD 4.5)
Vetrugno et al. (23)	19	Yes	15.8% (3/19)	15.8% (3/19)	From 15 to 2 years
Iranzo et al. (34)	26	Yes	53.8% (14/26)^#^	53.8% (14/26)^#^	–
De Cock et al. (25)	49	Rate of V-PSG-confirmed RBD not specified	30% (13/43)	26.5%	From 20 years to few months
Nomura et al. (26)	16	Yes	63.6% (7/11)	43.8% (7/16)	From 6 to 1 years^°^
Guo et al. (28)	30	Yes	21.4% (3/14)	10% (3/30)	1, 1.2, and 3 years
Stanzani-Maserati et al. (29)	10	Yes	60% (6/10)	60% (6/10)	From 3 to 1 years
Coon et al. (31)	685	No	34% (103/304)	15% (103/685)	–
Palma et al. (30)	64	Rate of V-PSG-confirmed RBD not specified	54.7% (29/53)^#^	45.3% (29/64)^#^	–
McKay and Cheshire (35)	30	V-PSG confirmed RBD in 2/5	25% (5/20)	16.7% (5/30)	–
Giannini et al. (32)	158	Yes	39.3% (42/107)	27% (42/158)	From 16 to 1 years, median 3 years (IQR 2–5)

*These prevalence rates were calculated from raw data, when not available in the studies*.

# *Prevalence of RBD onset before MSA onset was calculated considering only the motor onset (autonomic onset was not considered);*

° *latencies calculated from graphics; IQR, interquartile range; iRBD, isolated REM sleep behaviour disorder; RBD, REM sleep behaviour disorder; SD, standard deviation; V-PSG, video-polysomnography; SD, standard deviation*.

Among all studies investigating RBD predating MSA, two studies calculated prevalence and latency from RBD onset to disease onset taking into consideration only the motor onset and not the autonomic one (30, 34). Finally, some studies calculated prevalence on the overall sample to investigate the rate of iRBD as presenting feature of MSA, while others calculated this prevalence in MSA patients with RBD. Both values of prevalence and latency between RBD onset and MSA symptoms were reported in Table 2.

A retrospective study on 30 MSA patients sought to identify the very earliest symptoms in the natural history of disease and identified RBD as the third most frequent initial symptom of MSA (*n* = 5/30, 16.7%) after complete erectile failure and postural lightheadedness (35). Recently, a large monocentric study on 158 MSA patients compared disease onset according to the current international criteria (1) and considering RBD as a symptom of MSA onset revealed that this sleep disorder represents the most frequent first symptom of MSA. In this large cohort, patients with clinically suspected RBD had undergone V-PSG to confirm the diagnosis and, although the RBD onset was retrospectively reported by patients and caregivers, the event recording from V-PSG was shown to them to ensure it was the same reported in patient recall. Moreover, some MSA patients derived from the autonomic and sleep centres of the same Institute and were evaluated with closer follow-up before the onset of motor symptoms (32).

### Progression and Prognosis of MSA Patients With RBD as Initial Manifestation of Disease

Only one study investigated the disease progression and prognosis of MSA patients presenting with RBD (32). This study on 158 patients, 107 with V-PSG-confirmed RBD, showed that 42 patients presented RBD as first symptoms of MSA. Among patients with RBD predating MSA onset, 29 presented with history of autonomic failure (25 urinary symptoms, 6 symptomatic orthostatic hypotension), 17 with cerebellar syndrome, and 6 with parkinsonism (isolated or combined). The subgroups of patients with RBD preceding the disease onset showed a more rapid progression of disease. Comparing patients with RDB predating disease onset and those developing RBD after the disease onset, the former showed more frequently an autonomic onset and less frequently parkinsonism both at disease onset and during the disease course. Moreover, during the disease course, patients with RBD as presenting symptom revealed earlier onset of stridor, pyramidal signs, symptomatic orthostatic hypotension, and urinary symptoms and showed shorter latency of several milestones of disease progression (urinary catheterization, severe dysphagia, and wheelchair dependency). The risk of death estimated by Kaplan–Meier analysis was higher in patients with RBD before disease onset with a weak statistical significance (log-rank test, *p* = 0.05) (32).

### V-PSG Features in MSA With RBD

Concerning the clinical features of RBD in MSA, no differences were found between MSA-C and MSA-P subgroups (25, 27). As parkinsonism disappears during RBD movements in PD patients (36), the same research group investigated whether cerebellar symptoms and parkinsonism disappear during RBD in MSA patients. In this study, the authors interviewed 49 MSA and 49 PD patients along with their 98 bed partners, by means of a structured questionnaire, comparing movements, speech, and facial expressions during RBD and wakefulness. Clinical RBD was observed in 43/49 MSA patients. Reports from bed partners who were able to evaluate movements during sleep indicate that 81% of MSA patients reported some form of improvement during RBD compared to wakefulness. Movements were improved in 73% of patients with MSA, including increased speed (67%), strength (52%), or smoothness (26%). Speech was improved in 59% of patients and was more intelligible (17%), better articulated (36%), or louder in volume (55%). Facial expressions were normal (with disappearance of amimia and expressions of smiling, frowning, or fear) in 50% of patients during RBD. The rate of improvement was higher in PD than in MSA, but no further difference was observed either between MSA-P and MSA-C or in MSA patients with a disease duration <5 years vs. ≥5 years. Moreover, the analysis of movements was also monitored by V-PSG in 22/49 MSA patients and revealed more expressive faces and movements that were faster and more ample when compared with facial expression and movements during wakefulness. These movements were still somewhat jerky but lacked any visible parkinsonism, while cerebellar signs were not assessable. These findings suggest that there are still functional pathways in MSA patients (25).

Two studies investigated the longitudinal progression of V-PSG recordings in MSA patients with RBD. Decreased behaviours/movements during the disease course were confirmed on V-PSG recording in one patient. Sleep talking became less frequent and intense. At a more advanced stage, the ratio of RSWA to the whole of REM sleep increased, while the sleep architecture as well as the percentage of REM sleep were maintained (37). A V-PSG report on two MSA patients described a reduced frequency of RBD episodes during the disease course and the appearance of a disrupted sleep pattern characterised by no longer identifiable sleep stages, ambiguous and rapid oscillation of state-determining polysomnographic variables, associated with a nearly continuous motor and verbal abnormal behaviours. This disrupted sleep pattern was recorded after 4 and 6 years from RBD onset, and after 3 and 2 years from autonomic/motor onset, respectively. Features of abnormal Stage 1 NREM and REM sleep, together with unstable chin muscle tone, recurred rapidly and irregularly, in a sort of undifferentiated sleep state. These features were consistent with status dissociatus and were interpreted as progression of RBD (38).

## Phenoconversion to MSA in Patients With iRBD

Although almost all patients eventually diagnosed with MSA experience RBD at some point in their disease progression, studies investigating the rate of phenoconversion to synucleinopathies in cohorts of patients with iRBD showed that a low percentage of those who converted actually developed MSA. iRBD is a rare condition, and population-based studies showed a prevalence of about 1% over the age of 60. Few studies investigated the prevalence of RBD, using PSG, in population-based cohorts. One study on 348 individuals from South Korea aged 60 years and over quantified REM tone with PSG. Participants with abnormal tone were contacted by phone and asked about dream-enactment behaviour, revealing an RBD prevalence estimate of 1.15% (39). Another study estimated an RBD prevalence of 0.74% (95% CI = 0.29–1.89) in a sample of 539 individuals aged 60 years or older from a Spanish community, using a validated single question for the screening of RBD followed, in those who screened positive, by clinical assessment and V-PSG (40). Analysing data from 1997 participants belonging to the population-based HypnoLaus study (mean age = 59 ± 11.1 years, 53.6% women) who completed the Munich Parasomnia Screening questionnaire and had a complete polysomnography at home, authors estimated a prevalence of RBD of 1.06% (95% CI = 0.61–1.50) (41).

In the last years, several international groups have performed follow-up studies on iRBD cohorts focusing on the rate of phenoconversion to an overt neurodegenerative disease and on the predictive role of clinical and instrumental markers for phenoconversion.

In 1996, Schenck et al. first reported the phenoconversion of a series of 29 males older than 50 years of age, and in 2013, they provided a 16 year update of their previous report (42, 43). Other two important research groups closely followed up iRBD cohorts throughout the disease course. Since 2014, Postuma et al. recruited up to 154 patients with iRBD in Montreal (Canada), publishing subsequent articles on the longitudinal evolution of this cohort (44–48). Similarly, Iranzo et al., from Barcelona (Spain), first followed iRBD patients diagnosed from 1991 to 2003 and, subsequently, followed up disease-free patients from 2005 to date in a systematic manner, publishing articles on an increasing number of patients including up to 203 participants in this cohort (49–53). Moreover, other international groups focused on this topic and recently the International RBD Study Group combined prospective follow-up data, making up the largest iRBD cohort counting 1,280 participants recruited from 24 centres (54, 55). It is important to specify that this multicentre international group included overlapping patients with previous monocentric studies of smaller samples.

Taken together, these observational studies have suggested that most iRBD patients who eventually develop a defined neurodegenerative disease were almost always diagnosed with a synucleinopathy (43, 48, 53–63) (Table 3). These studies, generally performed in single centres and on small samples, reported a frequency of phenoconversion ranging from 12.5 to 80.8%. This variability in results could be related to the heterogeneity of methods and follow-up duration (43, 48, 53, 56–63). More recently, a larger study analysed prospective follow-up data on 1,280 iRBD patients from 24 centres of the International RBD Study Group, showing that 352 (28%) converted to an overt neurodegenerative syndrome (median time to phenoconversion = 8.0 years), 16 (4.5%) of which converted into MSA (55).

**Table 3:** Studies investigating phenoconversion in cohorts of patients with iRBD.

**References**	**Study sample**	**PSG**	**Overall follow-up of the study**	**Conversion risk**	**Phenoconversion**	**Prevalence of MSA in converters (%)**
Fantini et al. (56)	24 (75.0% males)	Yes	26.3 months (SD 5.0)	3 (12.5%) patients converted at follow-up	*n =* 2 PD	0.0
Boot et al. (57)	44 (77.3% males)	No	46 months (IQR 31–47)	15 (34.1%) patients evolved into neurodegenerative disease Median time between onset of dream-enactment behaviour and diagnosis of MCI/PD = 20.7 years	*n =* 1 PD *n =* 14 MCI	0.0
Wing et al. (58)	91 (82.4% males)	Yes	5.6 years (SD 3.3)	19 (20.9%) patients evolved into neurodegenerative disease The estimated 5 and 9 year risks of any neurodegenerative disorder for the overall study cohort were 8.5% and 38.1%.	*n =* 8 PD *n =* 8 AD *n =* 1 DLB *n =* 2 vascular dementia	0.0
Schenck et al. (43)	26 (100% males)	Yes	16 years	21 (80.8%) patients who were initially diagnosed with iRBD eventually developed parkinsonism/dementia Mean time to conversion = 14.2 ± 6.2 years (range: 5–29 years)	*n =* 13 PD *n =* 3 DLB *n =* 2 MSA *n =* 1 dementia (unspecified); *n =* 2, clinically diagnosed AD with autopsy-confirmed combined AD + Lewy body disease pathology.	9.5
Arnulf et al. (59)	69 (81.2% males)	Yes	3 years (range: 1–15)	16 (23.2%) converted into neurodegenerative disorders Median time from RBD onset to parkinsonism/dementia = 16 years	*n =* 6 parkinsonism *n =* 6 dementia *n =* 2 dementia plus parkinsonism *n =* 2 MSA	12.5
Mahlknecht et al. (60)	34 (85.3% males)	Yes	4.9 years (SD 0.3)	9 (26.5%) patients with iRBD developed Lewy body disease Mean interval from iRBD diagnosis to conversion = 5.5 ± 4.7 years	*n =* 6 PD *n =* 3 DLB	0.0
Fernández-Arcos et al. (53)	203 (79.8% males)	Yes	5.0 years (range: 0.1–17)	69 (34.0%) received a diagnosis of defined neurodegenerative syndrome after a median follow-up of 5 years	*n =* 22 PD *n =* 32 DLB *n =* 2 MSA *n =* 13 MCI	2.9
Youn et al. (61)	84 (69.1% males)	Yes	4.1 years (SD 2.1, range 1.0–10.3)	18 (21.4%) patients developed neurodegenerative disorders The estimated risk of developing neurodegenerative diseases was 9% at 3, 18% at 6 and 35% at 6 years from the diagnosis of iRBD, respectively.	*n =* 9 PD *n =* 4 DLB *n =* 1 MSA *n =* 3 AD *n =* 1 spinocerebellar ataxia	5.6
Li et al. (62)	43 patients (79.1% males)	Yes	5 years	18 (41.9%) developed neurodegenerative synucleinopathy diseases Median interval from the estimated onset of iRBD symptoms to conversion = 10.5 years	*n =* 9 PD *n =* 2 DLB *n =* 3 MSA *n =* 4 PD/MCI	16.7
Zhou et al. (63)	179 patients (79.1% males)	Yes	5.8 ± 4.3 years	50 (27.9%) patients developed neurodegenerative diseases Median time of conversion to neurodegenerative diseases = 9 years from RBD onset, 3.1 years from RBD diagnosis	*n =* 27 PD *n =* 7 DLB *n =* 2 MSA *n =* 14 AD	4.0
Fereshtehnejad et al. (48)	154 patients	Yes	8.2 years (SD 9.0)	55 (36%) converted to an overt neurodegenerative syndrome Mean interval between baseline evaluation and phenoconversion = 4.6 ± 2.5 years	*n =* 25 PD *n =* 4 MSA *n =* 26 dementia	7.3
Postuma et al. (54) (IRBDSG^*^)	279 (79.6% males)	Yes	3.8 years (SD 1.4)	93 (33.3%) developed a neurodegenerative disease Risk of neurodegenerative disease was 15% after 2 years, 25% after 3, 36% after 4, and 41% after 5 years Mean interval between baseline and disease diagnosis = 2.5 ± 1.7 years	*n =* 39 PD *n =* 7 MSA *n =* 47 dementia (*n =* 28 probable DLB)	7.5
Postuma et al. (55) (IRBDSG^*^)	1,280 patients (82.5% males)	Yes	3.6 years (max 19)	352 (27.5%) converted to an overt neurodegenerative syndrome Phenoconversion rate of 6.25% per year (10.6% after 2 years, 17.9% after 3 years, 31.3% after 5 years, 51.4% after 8 years, 60.2% after 10 years, and 73.5% after 12 years) Mean interval between baseline evaluation and phenoconversion was 4.6 ± 3.5 years	*n =* 199 parkinsonism (16 probable MSA) *n =* 153 dementia first.	4.6

*These prevalence rates were calculated from raw data, when not available in the studies*.

*AD, Alzheimer's disease; DLB, dementia with Lewy bodies; IRBDSG*

* *International REM Sleep Behaviour Disorder Study Group; n = sample; PD, Parkinson's disease; PSG, polysomnography; MCI, mild cognitive impairment; MSA, multiple system atrophy; SD, standard deviation*.

Considering only the sample who phenoconverted from iRBD, the rate of those who developed MSA ranged from 0% (56–58, 60) to 16.7% (62), with the remaining patients developing PD, DLB, or dementia (Table 3).

One retrospective study (64) focused on a subgroup of patients who experienced iRBD for at least 15 years before evolving into PD, PD dementia (PDD), DLB, or MSA. In 27 patients (88.9% males) with clinical history of RBD, 14 of whom with V-PSG-confirmed RBD, initial parkinsonian symptomatology was observed in 13 patients, initial cognitive impairment was reported by 13 patients, and primary autonomic symptomatology was the first symptom in one patient. At the last evaluation, the following diagnoses were established: MSA in 1 (3.7%) patient, PD in 4 (14.8%) patients, PDD in 6 (22.2%) patients, PD-MCI in 3 (11.1%) patients, MCI in 2 (7.4%) patients, and DLB in 11 (40.8%) patients. The median latency from RBD to neurodegenerative syndrome symptoms onset was 25 years (range 15–50 years) (64).

Few studies investigated predictors of phenoconversion in patients with iRBD. One recent study on the largest cohort of iRBD patients (*n* = 1,280) investigated potential predictors of phenoconversion identifying motor symptoms, objective motor examination, olfactory deficit, mild cognitive impairment, erectile dysfunction, abnormal DAT scan, colour vision abnormalities, constipation, RSWA, and age as markers (55). However, this study considered factors predicting the overall conversion rate from iRBD to an overt neurodegenerative disease, but not the ones specific for MSA conversion. A recent study analysed 154 polysomnography-proven patients with iRBD, of whom 55 phenoconverted to defined parkinsonism (*n* = 25 PD, n = 4 MSA) or dementia (n = 26 DLB) (48). Compared to the other synucleinopathies (DLB/PD), the four patients who eventually developed MSA were significantly younger (54.2 ± 7.8 vs. 70.4 ± 7.5, *p* = 0.001) and had preserved cognition (MoCA = 26.8 ± 2.5 vs. 21.3 ± 5.5, *p* = 0.044) and colour vision (FM100 = 87.0 ± 60.1 vs. 227.0 ± 122.9, *p* = 0.005) at the time of phenoconversion. MSA-converters may also have had less impaired olfaction (UPSIT % normal = 77.3 ± 44.4 vs. 57.6 ± 26.6) and more severe urinary symptoms (1.2 ± 0.4 vs. 0.7 ± 0.9) at phenoconversion (with marginal statistical significance levels) (48).

## RBD and Phenoconversion of Patients With IAF

Another pre-motor cohort is represented by patients with IAF, a synucleinopathy presenting with autonomic failure as the sole clinical manifestation that could convert to a manifest central nervous system synucleinopathy. As there is an overlap of autonomic dysfunction in patients with MSA and those with pure autonomic failure (PAF) (5 year history of IAF with normal neurologic examination), it may be challenging to differentiate these two disorders in early stages, when only IAF is present.

The presence of RBD in the context of autonomic failure was suggested to be confined to those evolving to MSA and to be absent in those with PAF in a previous case series, making RBD a clinical marker of MSA (65). However, subsequent case series have reported RBD in patients with PAF (66–68), with symptoms occurring after the onset of autonomic failure (68).

Disagreement in results may be a consequence of the differences in design, sample size, population characteristics, follow-up duration, and diagnostic criteria used in the studies.

Few studies on cohorts of patients with IAF have investigated the role of RBD in predicting phenoconversion.

The largest follow-up studies reported to date on the natural history of a cohort of patients with a 5 year history of IAF, who fulfil current criteria for PAF (*n* = 50, mean disease duration 13.8 ± 7.1), documented a global percentage of 32% of phenoconversion to a manifest synucleinopathy (converters *n* = 16, 10 with MSA). This study found that the latency of RBD onset, not its presence, allows the discrimination of the converters group. In fact, V-PSG-confirmed RBD, which was more frequent in the converters group without reaching statistical significance, occurred significantly earlier in this group during the disease course (2 [−2; 5] vs. 10 [4; 13] years, *p* = 0.0281). These data entail a higher risk of phenoconversion to other synucleinopathies in patients with IAF with early-onset RBD (hazard ratio = 8.05, *p* = 0.016) (16).

A multicentric prospective study on the natural history of 100 patients with IAF, 74 of those followed up longitudinally, reported a cumulative incidence of phenoconversion to central synucleinopathy of 34% (converters *n* = 25, 6 with MSA) during a limited 4 year follow-up period (≈14%/year). This study did not report RBD in the small group of patients retaining the PAF phenotype at the last follow-up (*n* = 12) and showed that the presence of RBD was strongly associated with phenoconversion to other synucleinopathies (14). In a monocentric retrospective cohort study on 318 patients (79 patients followed up for at least 3 years) with IAF, in which the estimated conversion rate ranged between 12 and 48% (38 of 318 and 38 of 79, respectively), 22 patients developed MSA. Conversely, compared to previous findings, RBD was not found to be associated with a higher risk of phenoconversion and was seen in about half of patients with stable PAF, but its presence or absence was not consistently documented (15).

However, the latter two studies included all patients with IAF, irrespective of the disease duration, with subtle non-specific neurologic deficits at the time of enrolment and followed patients up for a short period (4 year follow-up). Our previous study (16) included only patients with IAF with a disease duration of >5 years without subtle non-specific neurologic deficits at the time of enrolment who were followed up for a longer period, allowing better estimation of the phenoconversion rate in patients currently defined as having PAF.

## Conclusion

RBD is a key feature of MSA, playing a role in the presentation, prognosis, and progression of disease. Almost all MSA patients experience RBD at some point of the disease course, without differences in prevalence values between MSA-C and MSA-P groups. Although clinically suspected MSA is frequently reported in the majority of MSA patients, previous results demonstrated that V-PSG is needed to diagnose RBD, especially in those who do not report symptoms or do not have a sleep partner. Despite the high prevalence of RBD during the disease course, the rate of RBD as initial manifestation of MSA has been poorly investigated because only few studies primarily focused on this aspect. Prevalence of RBD as first symptom of disease onset ranged from 10% to 60.0% when calculated in overall MSA samples. One recent study (32), comparing the mode of MSA presentation according to the current international criteria (1) and to iRBD onset, reported that this sleep disorder represents the most frequent first symptom of MSA with a prevalence of 27%. Overall, data on the prevalence of RBD in MSA and of RBD as first symptom of disease argue for the inclusion of RBD as supportive feature of MSA diagnosis. The latest version of MSA diagnostic criteria in 2008 included additional features that allowed the diagnosis at an earlier stage in individuals who do not yet fulfil the requirements for “probable MSA” (1). Although the introduction of additional features led to improved diagnostic accuracy at the early disease stage (1), the overall sensitivity of these criteria remains suboptimal (69). This is related to the difficulty in distinguishing MSA from other more common synucleinopathies, especially at the early stages, but also from other mimics. Indeed, a recent study, performed on 203 cases, revealed that 160 (78.8%) patients were correctly diagnosed in life and had pathologically confirmed MSA, while the remaining 43 (21.2%) had alternative pathological diagnoses (70). For this reason, the introduction of RBD as supportive feature of MSA in the early disease stage could improve diagnostic accuracy.

Moreover, these studies suggest that RBD could be introduced as mode of MSA onset in forthcoming consensus criteria to evaluate the disease duration and also as a clinical feature of pre-motor and pre-autonomic presentation of MSA. Prodromal diagnostic criteria are required for MSA. Diagnostic criteria for prodromal MSA should optimise sensitivity in capturing an underlying alpha-synucleinopathy in patients presenting at a disease stage prior to that of “possible MSA” and should retain enough specificity to distinguish it from PD and DLB (13, 71). Prodromal diagnostic criteria for MSA could guarantee homogenous patient selection for clinical trials with therapies able to slow or prevent the neurodegenerative process.

However, although RBD is a robust marker of underlying alpha-synucleinopathy, its presence alone does not stratify among these different neurodegenerative disorders and other clinical and/or instrumental features in addition to RBD should be validated to increase diagnostic accuracy (e.g., neuroimaging, olfactory dysfunction, and central or peripheral autonomic failure).

Finally, patients who presented with RBD at disease onset showed a more rapid and severe disease progression, mainly due to a rapid involvement of autonomic features and to an early achievement of milestones of disease progression. These data entail the highly topographic and functional interconnection of brainstem neuronal networks, whose degeneration in MSA has been widely documented (72, 73), and suggest that a close follow-up is required to early recognise and treat stridor and autonomic disorders in this subgroup of patients.

## Author Contributions

GG: analysis of literature and drafting the manuscript. FP and PC: critical revision of the manuscript. GC-B: conception of the study and critical revision of the manuscript. All authors read and approved the final manuscript.

## Conflict of Interest

The authors declare that the research was conducted in the absence of any commercial or financial relationships that could be construed as a potential conflict of interest.
